# Does eating slowly influence appetite and energy intake when water intake is controlled?

**DOI:** 10.1186/1479-5868-9-135

**Published:** 2012-11-21

**Authors:** Ana M Andrade, Daniel L Kresge, Pedro J Teixeira, Fátima Baptista, Kathleen J Melanson

**Affiliations:** 1 Department of Nutrition and Food Sciences, 112 Ranger Hall, University of Rhode Island, Kingston, RI 02881, USA; 2 Exercise and Health Laboratory, Faculty of Human Kinetics, Technical University of Lisbon, Cruz-Quebrada, 1495-688, Portugal

**Keywords:** Eating rate, Water, Satiation, Appetite, Visual analogue scales, Energy intake regulation

## Abstract

**Background:**

Slow eating has been associated with enhanced satiation, but also with increased water intake. Therefore, the role of water ingestion in regard to eating rate needs to be discerned. This study examined the influence of eating rate on appetite regulation and energy intake when water intake is controlled.

**Methods:**

In a randomized design, slow and fast eating rates were compared on two occasions, in 30 women (22.7±1.2y; BMI=22.4±0.4kg/m^2^) who consumed an ad libitum mixed-macronutrient lunch with water (300 mL). Satiation was examined as the main outcome by measuring energy intake during meals. At designated times, subjects rated hunger, satiety, desire-to-eat, thirst, and meal palatability on visual analogue scales. Paired t-tests were used to compare hypothesis-driven outcomes. Appetite ratings were compared across time points and conditions by repeated measures analysis of variance (ANOVA) using a within-subject model.

**Results:**

Energy intake and appetite ratings did not differ between conditions at meal completion. However, subjects rated less hunger and tended to rate lower desire-to-eat and greater satiety at 1 hour following the slow condition.

**Conclusions:**

Results tend to support a role of slow eating on decreased hunger and higher inter-meal satiety when water intake is controlled. However, the lack of significant differences in energy intake under these conditions indicates that water intake may account for the effects of eating rate on appetite regulation.

## Background

Eating behaviors that promote excess energy intake may have contributed to the increases in the incidence of overweight and obesity [1]. For example, findings from population studies have shown that faster self-reported eating rates were associated with greater body mass index in Japanese individuals and in middle-aged women from New Zealand [2-4]. Altering eating behaviors to reduce the rate of eating has become a hallmark of many weight control programs [5,6], despite limited evidence supporting its effectiveness. In fact, a retrospective longitudinal study on this topic was recently published, and it showed that the fast-eating group of Japanese male workers had a higher average 8-year weight gain than the combined group of medium and slow eaters [7]. Similar findings have been earlier reported in fire fighters that reported eating faster at the station, whose weight gain over 7 years was 1.4 kg higher than all the others [8].

Slow eating has been hypothesized to help control energy intake since it allows satiation to register before too much food is consumed [1,9]. However, empirical evidence on this treatment approach is limited and has yielded inconsistent findings. Smaller bite sizes or pauses within meals have been associated with reduced energy intake in some studies [10,11], or conversely have led to no differences in overall meal intake [12,13].

These former studies have not directly manipulated eating rates and examined meal food intake between conditions using a within-subject design. Although an increased interest in eating rate has been noticed in recent years, studies of the effect of manipulating eating rates on energy intake have shown that slow eating reduced energy intake in some groups, but not consistently across studies [14-19]. For example, slower eating rate decreased food intake in men [16], but not in women [16], patients with bulimia nervosa [15], or highly restrained eaters [17].

Another study observed lower total energy intake and greater satiety after meal completion, with combined techniques of taking small bites, pausing between bites, and chewing thoroughly to alter eating rate, in healthy women [14]. However, eating slowly allowed time for the consumption of more water along with the meal, which led to higher total weight consumed. This may have increased stomach distension, and thus induced higher satiety [20]. Nonetheless, previous research has shown that drinking water with a meal, as opposed to the water incorporated into a food, does not necessarily affect energy intake [21].

Most previous studies of eating rate and energy intake have not reported fluid consumption, so the role of fluid intake in this relation needs to be clarified. Before slowing the pace of eating is recommended as a useful strategy to prevent overeating, the mechanisms underlying its effectiveness must be fully understood and nuances for practical strategies more clearly determined. To further validate the previous findings [14], the present study sought to compare the impact of slow and fast eating rates on energy intake and on the development of satiation in healthy women when water intake is controlled.

## Methods

### Participants

Thirty healthy, non-smoking, pre-menopausal females with body mass index (BMI) of 19-30kg/m^2 ^were recruited with flyers and classroom announcements on the University of Rhode Island campus. Exclusion criteria included allergies to test foods, caffeine or alcohol dependency, diabetes mellitus, adrenal or thyroid disease, any chronic illness that might cause weight change, clinically-diagnosed eating disorders, medications that might alter appetite, or dieting. Participants’ characteristics are shown in Table 1. The study was approved by the Institutional Review Board of the University of Rhode Island. BMI was cross-checked by the investigators at the laboratory before starting the study, and written informed consent was obtained from participants. By design, the purpose of the study was not fully disclosed to participants, since meal duration and food intake were covertly recorded. Participants were financially compensated for completion of the study.

**Table 1:** Subject characteristics

**Characteristic**	**Mean**	**SEM**	**Range**
Age (y)	22.7	1.2	18-45
Height (m)	1.7	0.0	1.5-1.9
Weight (kg)	62.4	1.7	44.9-91.9
Body Mass Index (kg/m^2^)	22.4	0.4	18.6-26.3
Waist circumference (cm)	79.7	1.8	67.5-100.0
% Body fat	26.2	1.3	17.5-38.5
H-P score ^2^	12.0	0.8	0-21
TFEQ Scores			
Dietary restraint score ^3^	10.1	0.8	1-20
Disinhibition score ^3^	6.5	0.6	1-12
Hunger score ^3^	6.8	0.5	2-14

n = 30 females^1^.

^1^ Self-reported eating rate: “Slow”: n=5; “Medium”: n=16; “Fast”: n=9.

^2^ Scores from the Herman-Polivy Questionnaire (H-P). Anchor score: 0-35 [23].

^3^ Scores from the Three-Factor Eating Questionnaire. Anchor score: restraint: 0-21; disinhibition: 0-16; hunger: 0-14 [24].

### Anthropometric measurements

During visit 1, body weight was measured in minimal clothing on a digital scale accurate to 0.1kg (BodPod, Life Measurements Inc., Concord, CA), height was measured to the nearest 0.1centimeter (cm) on a wall-mounted stadiometer (Seca 240, SECA, Hamburg, Germany) accurate to 0.1cm, and BMI (in kg/m^2^) was calculated. Waist circumference was measured (in cm) at the level of the umbilicus with a flexible tyvek measuring tape. Body composition was assessed after a minimum 2-hour fast by air displacement plethysmography (BodPod, Life Measurements Inc., Concord, CA) using standardized techniques [22].

### Questionnaires

Participants completed a personal health history questionnaire. Eating rate was self-reported as “slow”, “medium” or “fast”. Two validated instruments, the 10-item Herman-Polivy (H-P) Questionnaire, and the 51-item Three-Factor Eating Questionnaire (TFEQ), were also administered to assess levels of chronic weight-focused behavior, as well as dietary restraint, disinhibition, and hunger, respectively [23,24]. Participants completed the three questionnaires at home and investigators carefully checked all the questions with the participant when she arrived to the laboratory for visit 1.

### Study protocol

The next two laboratory visits were the test visits. A randomized crossover design was used to compare the experimental conditions of slow versus fast eating, with tests separated by 3–7 days. These visits were conducted during the mid-follicular phase of each participant’s menstrual cycle, to control for possible effects of the menstrual cycle on appetite [25]. Each test visit was conducted with one individual participant at a time. For the purposes of comparison, the study design was virtually identical to our previous protocol [14] but with controlled water intake instead of ad libitum water.

On the day prior to each test visit, participants were instructed to avoid strenuous physical activity, to refrain from alcohol and caffeine consumption, to maintain their diet as close to normal as possible (no extremes of nutrients and calories) and to match these conditions before both test days.

On the morning of test days, participants’ breakfasts were consumed at home, and were specifically prescribed (described below) and matched between the slow and fast eating conditions. Between breakfast and the lunch test, physical activity was minimized, and they were allowed to drink water in moderation (enough to quench thirst) but not more than 600mL. A graduated bottle was provided to each participant with the purpose of recording the volume of water consumed during the morning before each lunch test.

At lunch time, participants reported to the laboratory following a minimum 4-hour fast.

Prior to each test meal, participants were asked if they followed the instructions regarding breakfast intake, physical activity, and their diet on the day before, and their visit was rescheduled if they failed to follow the instructions. Participants began each lunch meal at the same time. After voiding, they were offered generous pre-weighed portions (690 grams) of a mixed-macronutrient lunch (described below) and 300mL of water (refrigerator-chilled). These quantities were based on previous work in our lab [14]. They were instructed to drink the water in its entirety throughout the meal, and to consume as much of the food as they would like, to the point of comfortable satiation. Under the fast condition, participants used a large spoon (soup spoon) and were instructed to consume the meal as fast as possible with no pauses between bites. However, they were instructed not to eat so fast that it was uncomfortable for them. During the slow condition, a small spoon (teaspoon) was provided with these meals. Participants were instructed to take small bites, put down the spoon between each bite and chew each bite 20 to 30 times. During both conditions, the investigator carefully monitored the participant, prompting her to eat according to protocol. Exact clock time of meal initiation and completion were covertly recorded. The amount of the meal consumed was calculated by weighed differences (to the nearest 0.01gram) using a digital scale (Adventurer, OHAUS Corp., Pine Brook, NJ), and eating rate was calculated as kcal/min and g/min.

### Assessment of appetite and meal palatability

Appetite was assessed with validated 10-cm visual analogue scales (VAS) [26]. For both conditions, hunger (VAS-H), satiety (VAS-S), desire-to-eat (VAS-D), and thirst (VAS-T) were assessed before test meal intake, every 5 minutes (min) during the meal up to 30min, again upon meal completion, and then at 45 and 60min. Participants were not allowed to eat or drink anything between meal completion and the final appetite ratings at 60min after meal initiation. Meal palatability was also assessed by VAS at 1min into each meal and after meal completion. These scales were anchored by the statements “not at all” and “extremely”.

### Test meals

On the morning of the two test days, participants consumed a standardized breakfast at home containing approximately 400kcals, consisting of 8oz of orange juice, 8oz of 1% or 2% milk, and 1 cup of ready-to-eat cereal (except Granola or Grape Nuts), with the option of consuming 8oz of decaffeinated tea or coffee. Participants consumed identical breakfasts on the morning before each test, and then fasted for a standard time (minimum 4hours) during which physical activity was minimized.

The test meal contained 1000kcals and consisted of ditalini pasta, canned diced tomatoes with Italian seasoning, tomato paste, celery and minced garlic sautéed in olive oil, and parmesan romano cheese. Ditalini pasta was chosen specifically because its small size allowed for slow or fast eating rates with small or large utensils. The macronutrient distribution of the test meal was 48% of energy from carbohydrate, 39% from fat and 13% from protein.

### Statistical analysis

The main study outcome was ad libitum energy consumption. Other outcomes included ratings of VAS-H, VAS-S, VAS-D, and VAS-T. Paired t-tests were used to compare energy intake, rate of energy consumption, and appetite ratings upon meal completion between fast and slow conditions. VAS-H, VAS-S, VAS-D, and VAS-T ratings were compared across time points and conditions by repeated measures analysis of variance (ANOVA) using within-subjects model. A square root transformation was applied to VAS appetite data prior to ANOVA because the data were not normally distributed. Violation of sphericity was corrected using the Box correction, and corrected degrees of freedom were reported. Significant results are reported, and the effect size is reported as partial eta squared (η^2^). The equation maximizing the time-by-condition interaction is reported (e.g. cubic model) for all significant ANOVA’s. *Post hoc* comparisons were performed using paired t-tests using Bonferroni correction [27]. Area under the curve (AUC) was calculated for ratings of appetite using the trapezoid method, and results were compared using paired t-tests. Meal palatability data were examined by paired t-tests. Data were also examined continuously by correlation analyses (Pearson’s tests) to determine the relationships of TFEQ and H-P scores with the main outcomes. Results are expressed as mean ± standard error of the mean (SEM), and were considered significant at p < 0.05. Data were analyzed using the software Statistica version 6.1 (StatSoft Inc., Tulsa, OK), and SPSS version 16.0 (SPSS Inc., Chicago, IL).

## Results

Thirty healthy, non-smoking, pre-menopausal females were recruited and all completed the study (Table 1). The majority of participants were college students. Thirteen women scored higher than 10 in the TFEQ-restraint, which is common in this population. However, neither H-P nor TFEQ scores correlated with the ad libitum lunch variables (p > 0.05).

There was no difference in the amount of water consumed during the morning before participants’ arrival at the laboratory for the lunch test (fast: 375.3 ± 30.9 mL; slow: 347.2 ± 33.9 mL; p > 0.05). As shown in Table 2, meal duration was approximately 18min longer under the slow condition. However, there was no significant difference in the weight of food consumed and therefore no difference in energy intake. Since water intake was controlled, all participants consumed 300 mL of water. The combination of small bites, pauses between bites, and thorough chewing during the slow condition resulted in a significantly decreased eating rate, expressed as kcal/min. Participants consumed 70.8 ± 2.6% of the available energy presented at the meal under the fast condition and 69.4 ± 3.3% under the slow condition (p > 0.05). No participant consumed the full amount of food offered under either condition.

**Table 2:** **Meal duration**, **ad libitum meal intake**, **and eating rate in the two experimental conditions** (**fast and slow**)

	**Experimental condition**^**1**^				**Number of subjects**	
	**Fast**		**Slow**		**Fast > Slow**^**2**^	**Slow > Fast**^**3**^
	**Mean**	**SEM**	**Mean**	**SEM**	**n**	**n**
Duration of the meal (min)	8.4	0.6	26.1	1.8 *	0	30
Weight of food consumed (g)	488.2	17.9	478.6	22.5	17	13
Energy intake (kcal)	707.9	26.0	694.0	32.6	17	13
Energy density (kcal / total g)	0.9	0.0	0.9	0.0	17	13
Rate of energy consumption (kcal/min)	94.0	5.6	29.0	1.9 *	30	0

n = 30 females.

^1^ Statistical differences between conditions were determined by paired t-tests: * p < 0.05.

^2^ Number of subjects in which meal duration and intakes were higher in the fast condition than the slow.

^3^ Number of subjects in which meal duration and intakes were higher in the slow condition than the fast.

**Figure 1:**
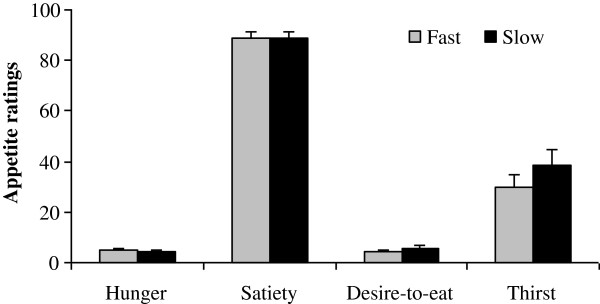
Visual analogue scale appetite ratings (mean ± SEM) upon meal completion from thirty women who consumed an identical ad libitum meal and 300mL water under fast and slow eating conditions, in randomized order.

VAS-H, VAS-S, VAS-D and VAS-T ratings upon completion of the ad libitum meal consumed under fast and slow conditions are shown in Figure 1. VAS-H, VAS-S, and VAS-D ratings did not differ between conditions immediately after finishing the meal (p > 0.14) while VAS-T ratings tended to be higher immediately upon completion of the meal consumed at a slow rate (p = 0.09).

**Figure 2:**
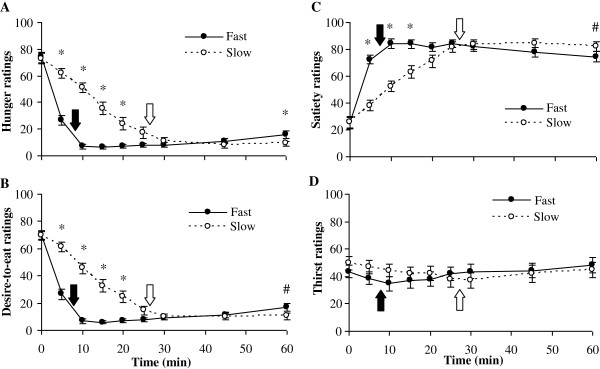
**Visual analogue scale appetite ratings over time (mean ± SEM), from thirty women who consumed an identical ad libitum meal and 300mL of water under fast and slow eating conditions, in randomized order: A–Hunger; B–Desire-to-eat; C–Satiety; D-Thirst.** Closed and open arrows represent meal completion for the fast (~8min) and slow (~26min) conditions, respectively. All appetite ratings, except thirst, showed a significantly time-by-condition interaction effect. AUC for hunger and desire-to-eat was significant different between conditions. * Means at given time points were significantly different between conditions (p < 0.006). ^#^ Desire-to-eat (p = 0.008) and satiety (p = 0.017) ratings were marginally significant different at 60min.

The effect of eating rate on ratings of VAS-H, VAS-S, VAS-D and VAS-T over time is represented in Figure 2. The open and closed arrows represent average time of meal termination for the fast (8min) and slow (26min) conditions, respectively. There was a significant effect of eating rate on hunger (Linear model: F_[1,29]_ = 32.72, p < 0.001, η^2^ = 0.530). There was also a significant effect of time on hunger (Linear model: F_[1,29]_ = 241.270, p < 0.001, η^2^ = 0.893), and a significant time × eating rate interaction effect on hunger (Cubic model: F_[1,29]_ = 68.21, p<0.001, η^2^ = 0.702). *Post hoc* testing revealed that in the slow condition, hunger was significantly higher at 5, 10, 15, and 20min (p < 0.001) and was significantly lower at 60min (p = 0.003) compared with the fast condition, as shown in Figure 2A. AUC for hunger in the fast condition (824 ± 87 mm·min) was significantly lower (t = 5.052, p < 0.001) than the slow condition (1431 ± 132 mm·min). There was a significant effect of eating rate on desire-to-eat (Linear model: F_[1,29]_ = 39.23, p < 0.001, η^2^ = 0.575). There was also a significant effect of time on desire-to-eat (Linear model: F_[1,29]_ = 188.94, p < 0.001, η^2^ = 0.867), as well as a significant time × eating rate interaction effect (Cubic model: F_[1,29]_ = 77.73, p < 0.001, η^2^ = 0.728). *Post hoc* testing revealed that in the slow condition, desire-to-eat was significantly higher at 5, 10, 15, and 20min (p < 0.001) and tended to be lower at 60min (p = 0.008) compared with the fast condition, as shown in Figure 2B. AUC for desire-to-eat during the fast condition (834 ± 81 mm·min) was significantly lower (t = 5.337, p < 0.001) than the slow condition (1391 ± 126 mm·min). There was a significant effect of eating rate on satiety (Linear model: F_[1,29]_ = 10.63, p = 0.003, η^2^ = 0.268) and a significant effect of time on satiety (Quadratic model: F_[1,29]_ = 123.75, p < 0.001, η^2^ = 0.810), as well as a significant time × eating rate interaction effect (Cubic model: F_[1,29]_ = 36.82, p < 0.001, η^2^ = 0.559), as shown in Figure 2C. *Post hoc* testing revealed that in the fast condition, satiety was significantly higher at 5, 10, and 15min (p < 0.001) and tended to be lower at 60min (p = 0.017) compared with the slow condition, as shown in Figure 2C. AUC for satiety in the fast condition (4643 ± 175 mm·min) was marginally higher (t = 2.018, p = 0.053) than the slow condition (4315 ± 159 mm·min). There was no significant effect of eating rate, time or time × eating rate interaction effect on thirst, as shown in Figure 2D. AUC for thirst in the fast condition (2522 ± 280 mm·min) was not significantly different (t = 0.026, p = 0.979) than the slow condition (2521 ± 295 mm·min).

Palatability ratings for the ad libitum meal did not differ significantly between conditions at the beginning or after consumption. Pleasantness and tastiness ratings were approximately 80%, suggesting participants found the meal to be highly palatable. Ratings of saltiness were approximately 30% in both conditions (data not shown).

## Discussion

The results of this study show that when water intake is controlled, combining the techniques of small bites, pauses between bites, and chewing thoroughly is associated with decreased eating rate, less hunger, and higher satiety, but not with decreased energy intake. This has interesting implications for recommending these techniques in the context of behavioral energy intake regulation. Eating slowly in order to reduce energy intake and enhance satiation appears to be most effective when ad libitum water is served with the food [14]. Martin et al. [16] also reduced participants’ habitual rate of intake, which led to decreased food intake in men but not in women. They served ad libitum water with the meals, but there was no difference in water intake, so eating slowly may not necessarily lead to drinking more water. However, eating rate in that study was slowed by having participants take a bite of food when prompted by a computer. Although the present study also took place in a laboratory setting, eating rate was manipulated in a more natural manner. Actually, thirst ratings at meal completion suggest that participants might have consumed more water under the slow condition if it was available. Water consumption may play an important role on the effects of eating rate on food intake. However, water intake was not reported in the majority of the studies on eating rate, or was controlled but with contradictory results [17,18], so empirical evidence must be provided before precise conclusions can be made with respect to synergies between eating rate and water intake.

In a previous study, thirty women with similar characteristics to the women in this study consumed the same pasta meal with water consumed ad libitum as the only aspect of the protocol that differed from the present study [14]. Under the slow condition, participants consumed significantly less food than during the fast condition. However, weight of water and total weight consumed were significantly higher in the slow condition [14]. In contrast with those findings, the present study’s lack of an effect of eating rate on food intake when water intake is matched suggests that the lower energy intake previously verified may not be attributable simply to a slower eating rate. Peripheral satiety signals associated with meal termination include measures related to stomach distension, gastric emptying rate, and responses of several hormones, such as cholecystokinin, peptide YY and glucagon-like peptide 1 [20]. It has been proposed that slowing the rate of ingestion allows more time for these processes to take place, lengthening the satiety curve, and reducing total energy intake [9]. Two recent studies have explored this hypothesis, by measuring the postprandial gut peptide responses following fixed-portion meals consumed at different eating rates, and the results differed from one another [28,29]. Since these physiological signals were not measured in the current study, a relationship between food intake and the time course of post-ingestive meal termination signals cannot be established. Nevertheless, the present findings suggest that, when the water was served ad libitum, the physiological satiety signals could have been modulated by the larger quantities of water consumed during the slow condition [14]. It is possible that this might have increased stomach distension, and thus induced greater satiation. Laboratory studies testing direct manipulations of the volume in the stomach support this hypothesis [30].

Water has a major influence on energy density because it contributes weight without adding energy to foods. Rolls, et al. [21] showed that water incorporated into the food decreases energy intake, but water served as a beverage has little effect on overall energy intake. In this paradigm, the authors suggested that water in food probably suppresses hunger due to increases of weight and volume of the food and to changes in the dispersion of nutrients, while water consumed with the food would affect thirst [21], which was recently confirmed by Martens and Westerterp-Plantenga [31]. In agreement, while meal energy density affected meal intake in free-living adults, this response was not modulated by the addition of drinks to those meals [32]. Drinks added to a meal might therefore not be expected to affect overall consumption by a “diluting” mechanism. However, results from the two studies analyzing eating rate and water intake (ad libitum [14] and controlled) showed that drinking water with a meal may also play a role on food intake and satiation.

Few studies have directly addressed the hypothesis that water ingestion may modulate appetite within meals. The consumption of water with a meal has shown little effect on food intake, but mean ratings of fullness after the meal were higher than the no-beverage condition [33]. In contrast, drinking water during a meal reduced subjective ratings of hunger and increased satiety, but this effect was not maintained after the meal [34]. Nonetheless, Martens and Westerterp-Plantenga [31] have lately explored this hypothesis and showed that drinking water separately with a meal vs. water consumed in the food mainly quenches thirst, but hunger is not affected. Population-based studies have also shown that daily energy intake of water consumers is lower than that of non-water consumers [35], suggesting that water consumption may play a role on energy intake and possibly body weight regulation.

Despite similar ad libitum food intake when water intake was matched, appetite ratings at 60min show a potential benefit of slow eating. As shown in Figure 2, participants rated less hunger and tended to rate lower desire-to-eat and greater satiety at 60min following the slow condition. These trends support the hypothesis that changing eating behavior might have the ability to alter physiological satiety signals. Prolonging meal ingestion may increase the time of exposure of nutrients to gastrointestinal signals and this is independent of the amount of food eaten [9]. This suggests that satiation at subsequent meals or snacks could have been affected by slow eating, which is of particular interest for future analyses. More studies are needed on the effect of eating rate on inter-meal satiety and subsequent meals, since available research is limited and inconsistent [28,36].

Strengths of this study include the careful control of activity and of food and fluid intake before the tests, and testing only during the mid-follicular phase of the participants’ menstrual cycle. Limitations include the limited range of participant characteristics. Future studies should explore these hypotheses in different populations, such as males, as evidence shows that eating rate and its effects may differ by gender [4,16]. Moreover, results require further replication in real life conditions and with different foods and beverages.

## Conclusions

Although prospective studies are needed to corroborate the present findings, these data, together with earlier findings, suggest that recommendations directed at changing eating behavior for the purpose of energy intake reduction should be made carefully. The present evidence is consistent with the possibility that advising people to eat more slowly, take smaller bites, and chew their food thoroughly is generally helpful for appetite regulation, but especially when water is available for ad libitum consumption. Overall, allowing time to drink sufficient quantities of water along with a given meal while slowing down eating rate appears to be the most advisable strategy to maximize satiation and regulate energy intake.

## Competing interests

PJT is a member of the scientific committee of the Institute for Hydration and Health (Portugal) for which he receives no financial or other compensation. All other authors declare that they have no competing interests.

## Authors’ contributions

KJM was responsible for the conception, design and direction of the project. AMA collected the data and wrote the first draft of the manuscript. AMA and DLK performed the statistical analysis. PJT and FB reviewed the entire manuscript and made important contributions to various sections. All authors provided significant advice, contributed to and approved the final manuscript.
